# Studies on endoscopic submucosal dissection in the past 15 years: A bibliometric analysis

**DOI:** 10.3389/fpubh.2022.1014436

**Published:** 2022-09-27

**Authors:** Deqing Wu, Mengyu Jia, Shu Zhou, Xiaorong Xu, Meiqin Wu

**Affiliations:** ^1^Department of Gastroenterology, Shanghai Tenth People's Hospital, Tongji University School of Medicine, Shanghai, China; ^2^Shanghai Key Laboratory of Maternal Fetal Medicine, School of Medicine, Shanghai Institute of Maternal-Fetal Medicine and Gynecologic Oncology, Shanghai First Maternity and Infant Hospital, Tongji University, Shanghai, China

**Keywords:** endoscopic submucosal dissection, bibliometric analysis, Web of Science, data visualization, CiteSpace

## Abstract

**Background and aims:**

Endoscopic submucosal dissection (ESD) is an advanced minimally invasive technique for en bloc resection of superficial gastrointestinal lesions, which is drawn an increasing attention from its emergence. This bibliometric analysis is to evaluate the origin, current hotspots, and research trends on ESD.

**Methods:**

A total of 2,131 publications on ESD from 2006 to 2020 were obtained from the Web of Science Core Collection (WoSCC) database. Bibliometric visualization analyses of countries/regions, institutes, authors, journals, references and keywords were performed by CiteSpace V.5.8.R3.

**Results:**

The quantity of publications on ESD increased significantly during the past 15 years. Japan occupied the leading position in terms of research power. Professor Yutaka Saito, together with the institute he belongs, the Endoscopy Division, National Cancer Center Hospital, Tokyo, Japan, were the most productive author and institute, respectively. Colorectal ESD led the main thematic concentrations in ESD research. The most prolific journal was Gastrointestinal Endoscopy. European ESD Guideline was the most frequently co-cited reference. Guideline, meta-analysis, endoscopic resection, poly-glycolic acid sheet, Barrett's esophagus, fibrin glue, risk and colorectal neoplasm will be the active research hotspots in the future.

**Conclusions:**

These findings provide the trends and frontiers in the field of ESD, as well as valuable information for clinicians and scientists to discover the future perspectives with potential collaborators.

## Introduction

Endoscopic submucosal dissection (ESD) is an endoluminal surgical technique initially developed for early gastric cancer (EGC) in Japan in the late 1990s and early 2000s (1–3). Since its advantage in facilitating the en bloc resection and allowing precise histological staging of superficial tumors, ESD has been mainly applied to the treatment of early cancers and large lesions in gastrointestinal tract (4–8). The therapeutic effect and feasibility of this minimal invasive technique has been well–established by a series of data (9–14). Although it has been recognized as a mature endoscopic technique, the development of ESD is unbalanced between Eastern and Western countries due to various reasons, such as limited number of experts and the differing prevalence of gastrointestinal luminal diseases (15). The vast majority of experience and guidelines for ESD comes from Japan. Nevertheless, experience with ESD and evidence on its safety and efficacy have accumulated in Europe and the United States over the past decade (16–19). However, so far there lacks systematic research on global research trends and hotspots in this field.

Bibliometric research is a statistical and quantitative analytical method designed to identify the characteristics of publications and academic impact of journals, researchers, institutions, and countries within a research field by Dr. Chaomei Chen (20), which can help researchers detect the trends and identify frontiers in a certain research field (21–25). Related studies such as endoscopic retrograde cholangiopancreatography (ERCP) (26) and endoscopic ultrasound (EUS) (27) were using this method. However, no bibliometric analysis on ESD has been reported to date.

In the present study, we collected and screened the literatures on ESD in a 15-year interval (2006-2020) based on the Web of Science (WoS) database. CiteSpace was applied to investigate the global trends and the research frontiers in these publications by a visualized way. The purpose of this study is to clearly explore the origin and major milestones of the research on ESD, providing references for future research direction and cooperation.

## Materials and methods

### Data source

The retrieval data for the statistical analysis were screened from the Web of Science Core Collection (WoSCC), which providing citation search, giving access to multiple databases that reference cross-disciplinary research and allowing for an in-depth exploration of specialized subfields (21).

### Search strategy

All data were obtained from WoSCC on Dec 13, 2021. And the data retrieval strategy was as follows: (i) Title = endoscopic submucosal dissection or esophageal ESD or gastric ESD or colorectal ESD or duodenal ESD. (ii) Document type = article and review. (iii) Language = English. (iv) Timespan (custom year range) = 2006 to 2020. Full records and cited references were selected as a plain text format and downloaded for further analysis.

### Analysis tool

CiteSpace V.5.8.R3 was selected to perform the bibliometric analysis on the publications related to ESD by integrating information about countries/regions, authors, institutes, journals, citation, and keywords, aiming to provide scientific and intuitive support for clinicians and researchers in this field. CiteSpace, which was created by Dr. Chaomei Chen (School of Information Science and Technology, Drexel University, Philadelphia, PA, United States) and his team in 2004 (28), is a Java application which combines information visualization methods, bibliometrics, and data mining algorithms in an interactive visualization tool.

### Data analysis

The datasets for the analysis of publications on ESD were developed as a test platform for CiteSpace. The time span was from January 2006 to December 2020, which was sliced into 5 parts corresponding to 5 different colors, each of which was 3 years. The analyses of the cooperation networks (including countries/regions, authors, institutes and journals), reference co-citations, keywords co-occurrence cluster analysis and keywords burst detection were performed. The results were visualized by different types of clusters in a node-circle network according to the type of analysis. The nodes represent the analyses objects. The color and thickness of circles in each node indicate frequencies in different time period. The bright purple outer circles indicate the centrality, which is an index for measuring the importance of a node in a network, and high centrality is typically regarded as a pivotal point in a field (22). Keywords burst detection based on Kleinberg's algorithm was used to obtain future research direction (29).

## Results

### General data

**Figure 1:**
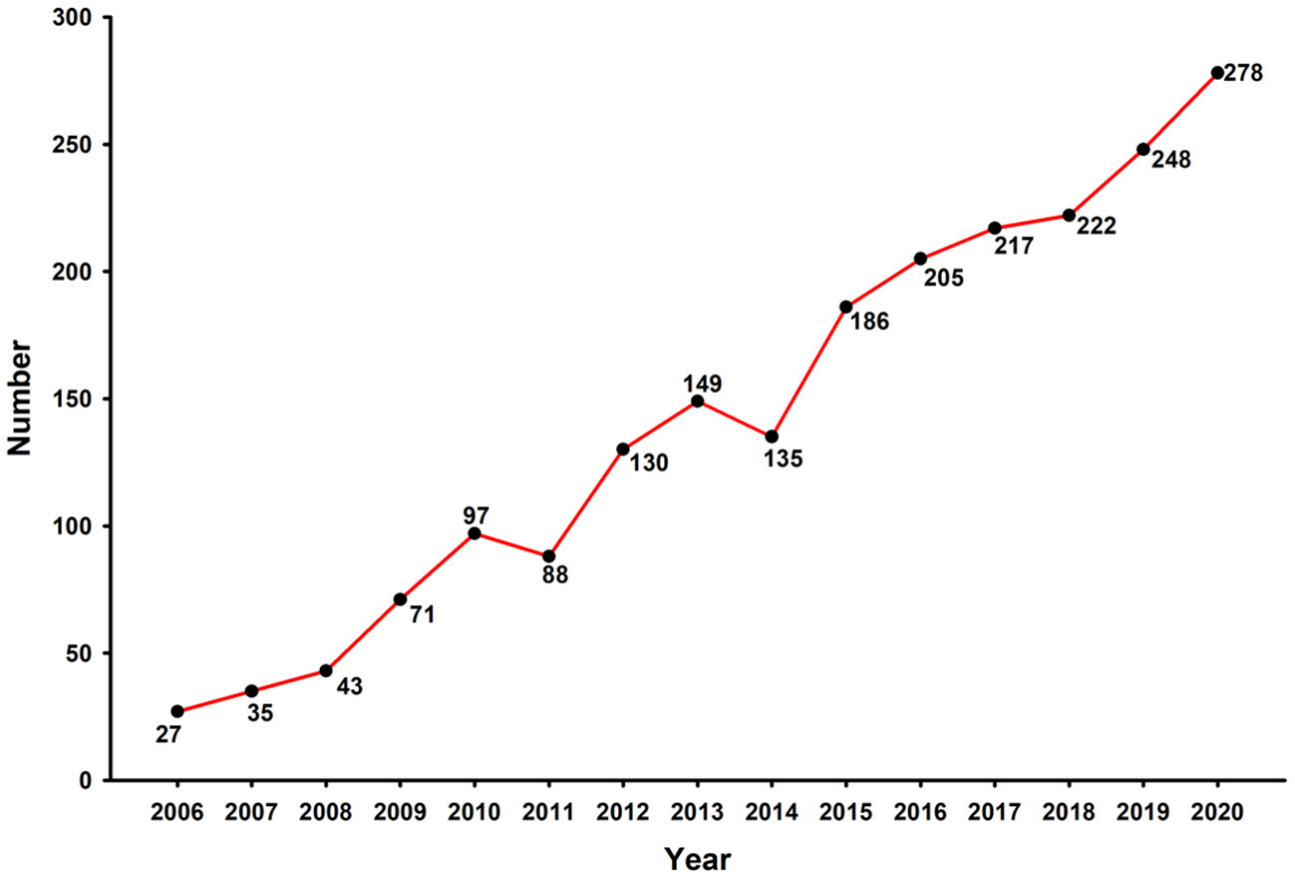
The number of ESD publications indexed by WoSCC, 2006-2020.

The search strategy for ESD generated 2,131 literatures, including 1,936 original articles and 195 reviews, published in English between 2006 and 2020, after filtering out the duplicate records. According to the publication years, the quantity of published articles on ESD increased significantly from 27 in 2006 to 278 in 2020, with an average annual growth rate of 66.40% (Figure 1).

### Countries/regions

**Figure 2:**
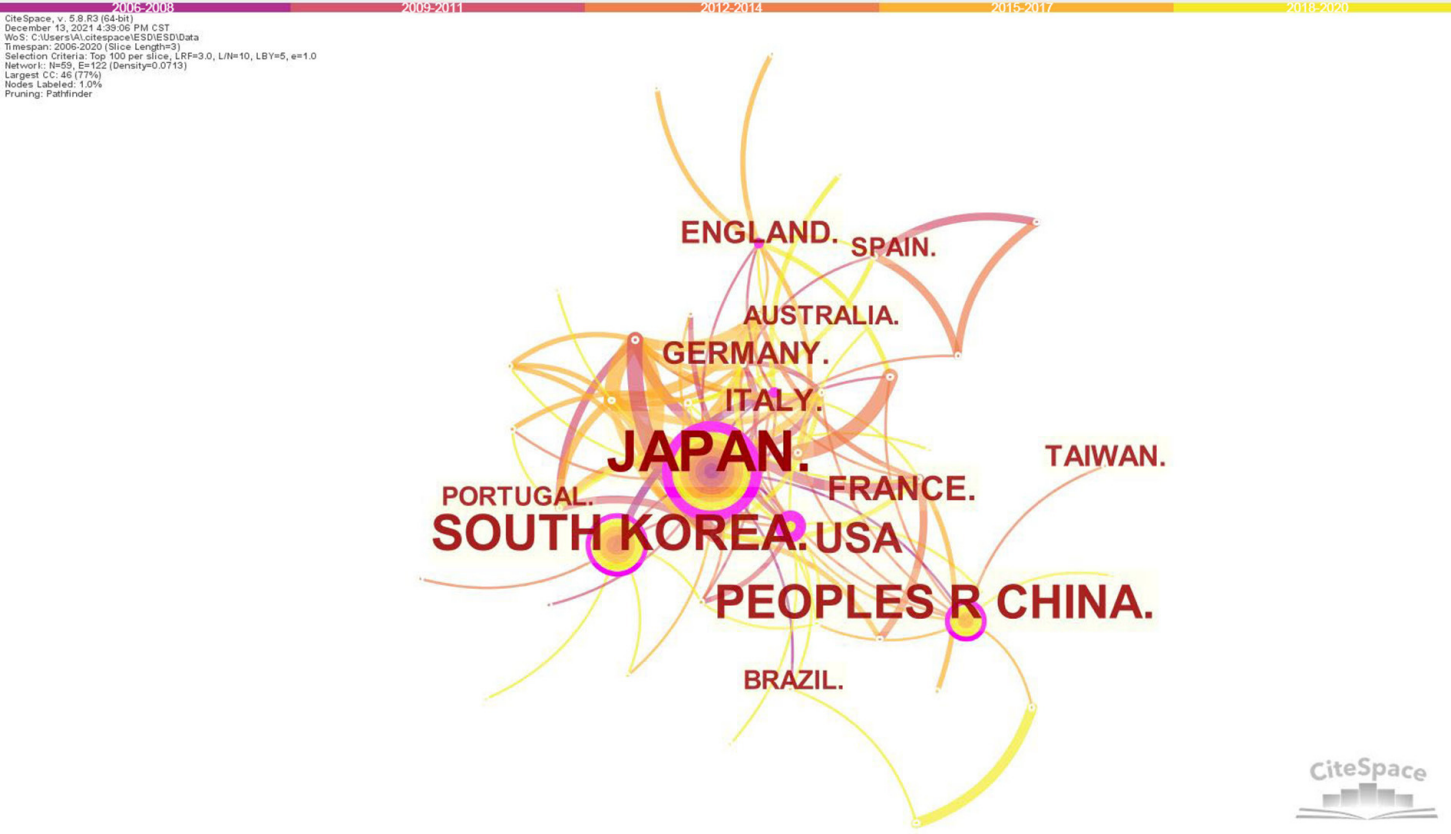
Map of countries/regions cooperative relations in research of ESD, 2006-2020. The bigger the circle, the more original articles the country/region published. The shorter and thicker the connection line, the closer the relationship between countries/regions.

The network of the productive countries/regions was shown in Figure 2. The size of circles represents the number of publications of countries/regions, and the shorter distance between two circles suggests the more collaboration between individual countries/regions. A circle with a wider purple ring indicates higher centrality, which is typically regarded as the pivotal point of a field. Among all relevant countries/regions, Japan (1,131) ranked first in the publication quantity, which was followed by South Korea (362) and People's Republic of China (290). The top 10 prolific countries/regions in the research of ESD were shown in Table 1. Countries/regions form Eastern Asia accounted for the majority (85.08%) of the publications on ESD. The United States led both the quantity and centrality of publications among western countries.

**Table 1:** Top 10 prolific countries/regions in research of ESD, 2006-2020.

**Ranking**	**Country/region**	**Frequency**	**Centrality**
1	JAPAN	1,131	0.37
2	SOUTH KOREA	362	0.12
3	People's Republic of China	290	0.19
4	United States of America	136	0.30
5	France	50	0.08
6	Germany	42	0.06
7	Italy	41	0.16
8	England	38	0.15
9	Taiwan	30	0.00
10	Australia	21	0.03

### Institutes

**Figure 3:**
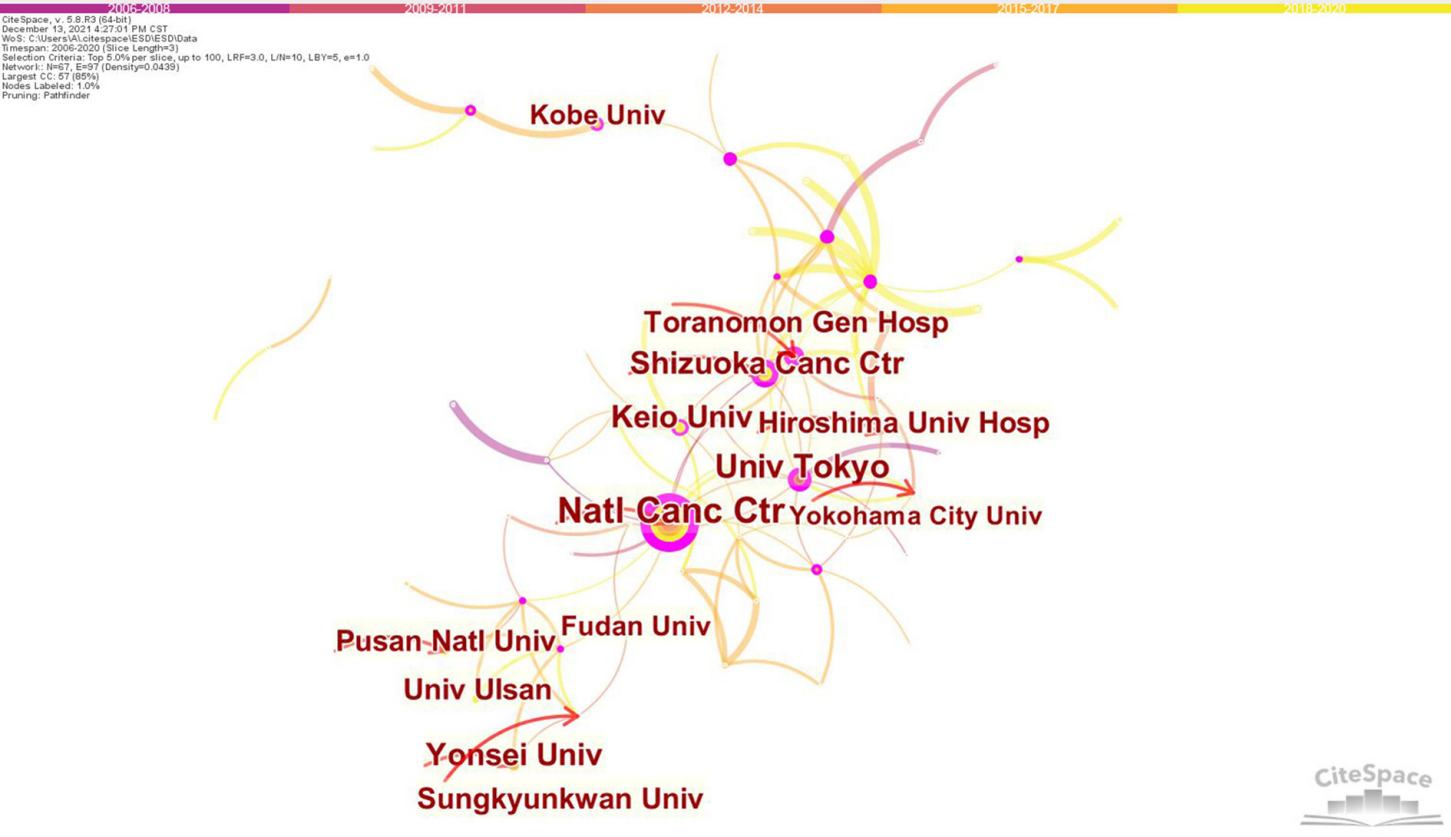
Map of institutes cooperative relations in research of ESD, 2006-2020. The bigger the circle, the more original articles the institute published. The shorter and thicker the connection line, the closer the relationship between institutes.

Figure 3 showed the major productive co-institutes in the field of ESD. The National Cancer Center hospital, Tokyo led the most productive and influential institutes in this field, with a total number of 134 published articles, followed by University of Tokyo (76 publications) and Shizuoka Cancer Center (67 publications). Notably, 7 out of the top 10 prolific institutes belonged to Japan, suggesting the country had a dominant position in the current researches on ESD (Table 2).

**Table 2:** Top 10 prolific institutes in research of ESD, 2006-2020.

**Ranking**	**Institute**	**Frequency**	**Centrality**
1	National cancer center hospital, Tokyo	134	0.45
2	University of Tokyo	76	0.30
3	Shizuoka cancer center	67	0.34
4	Yonsei university	60	0.00
5	Keio university	55	0.19
6	Toranomon gen hospital	52	0.31
7	University of Ulsan college of medicine	47	0.00
8	Pusan national university	46	0.00
9	Sungkyunkwan university, Seoul	45	0.01
10	Hiroshima university Hospital	45	0.06

### Authors

**Figure 4:**
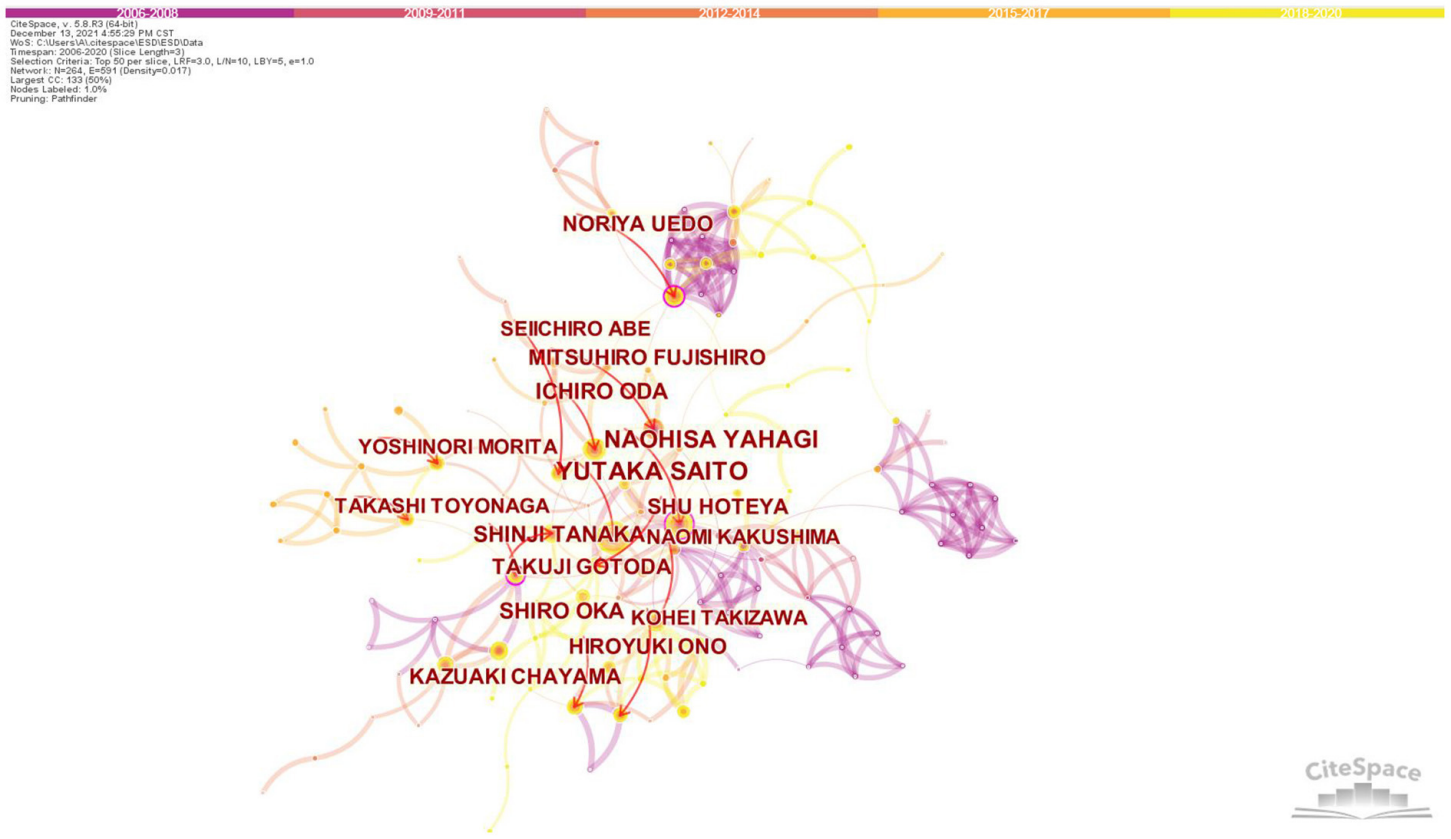
Co-authorship network map in research of ESD, 2006-2020. The bigger the circle, the more original articles the author published. The shorter and thicker the connection line, the closer relationship between authors.

For the identification of potential cooperation between authors, the co-authorship was illustrated by a network map generated by CiteSpace (Figure 4). Cooperation relationships are represented by connections among nodes. The thicker the connection is, the closer the cooperation is. Regarding the authors who were active, Yutaka Saito from the Endoscopy Division, National Cancer Center Hospital, Tokyo, Japan, ranked the first (77 publications), followed by Naohisa Yahagi from the Division of Research and Development for Minimally Invasive Treatment, Cancer Center, Keio University School of Medicine, Tokyo, Japan (64publications). Table 3 showed the top 10 prolific authors, who all came from Japan.

**Table 3:** Top 10 active authors in research of ESD, 2006-2020.

**Ranking**	**Author**	**Institute**	**Publications**	**Centrality**
1	Yutaka Saito	National cancer center hospital, Tokyo	77	0.05
2	Naohisa Yahagi	Keio university	64	0.16
3	Ichiro Oda	National cancer center hospital, Tokyo	52	0.07
4	Shiro Oka	Hiroshima university hospital	50	0.01
5	Shinji Tanaka	Hiroshima university hospital	50	0.11
6	Hiroyuki Ono	Shizuoka cancer center	47	0.01
7	Takuji Gotoda	Nihon university school of medicine, Tokyo	44	0.04
8	Kazuaki Chayama	Hiroshima university hospital	42	0.03
9	Noriya Uedo	Osaka international cancer institute	41	0.11
10	Shu Hoteya	Toranomon hospital, Tokyo	41	0.03

### Reference co-citation

**Figure 5:**
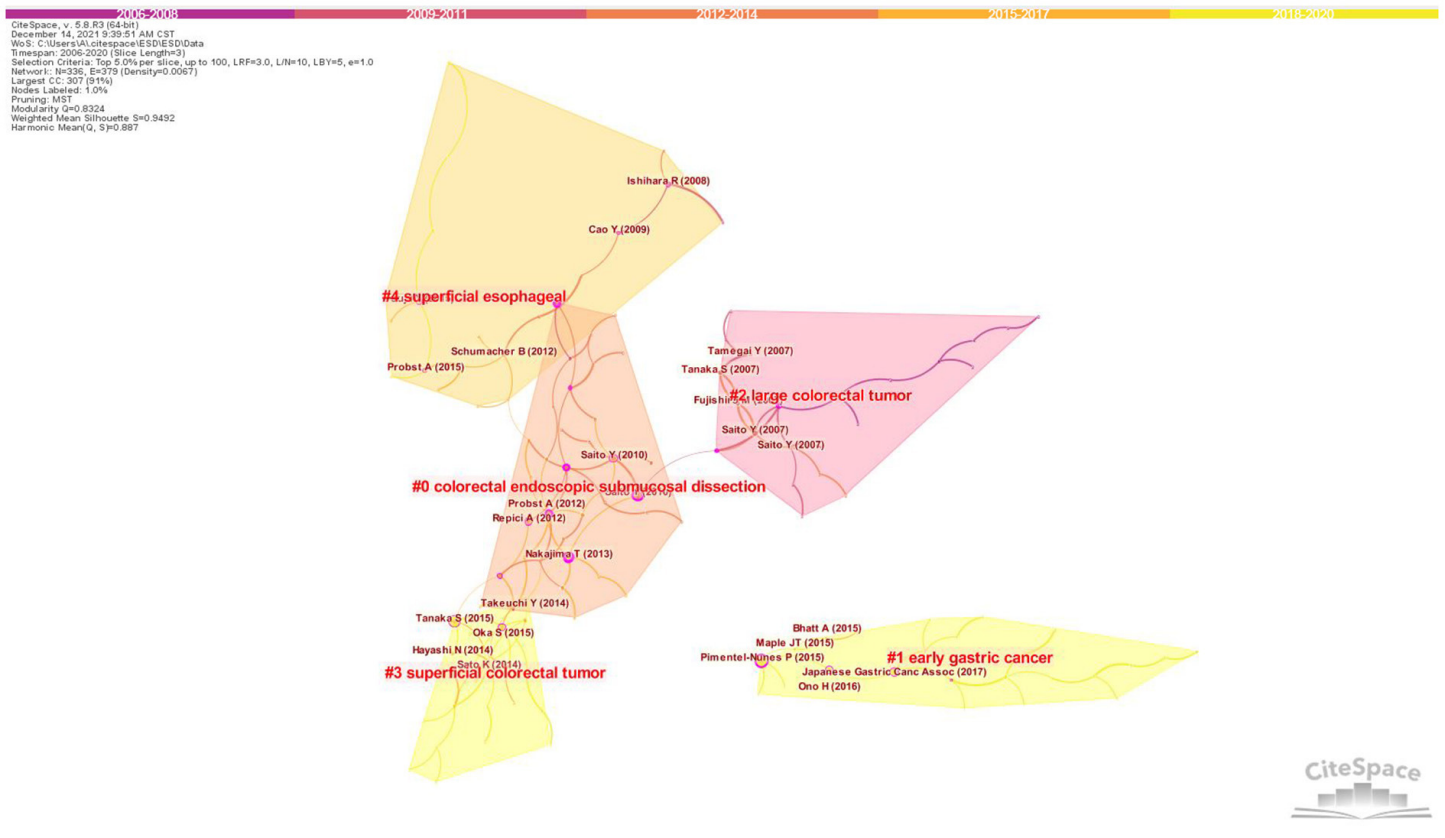
Clustering map of reference co-citation related to research of ESD, 2006-2020. The larger the circle, the more frequently it is co-citated. The wider the purple circle, the stronger the centrality.

Visualization of the largest reference co-citation network was shown in Figure 5, which was divided into 5 major co-citation clusters. These clusters were named by index terms extracted from the titles of the cited articles. The nodes represent different cited references and the clusters represent main thematic concentrations in ESD research. The top ranked cluster was colorectal endoscopic submucosal dissection, followed by early gastric cancer and large colorectal tumor. Table 4 showed the summary of the top five clusters.

**Table 4:** Summary of the largest 5 clusters.

**Cluster ID**	Size^*^	**Silhouette^#^**	Label (LLR^**^)	**Mean**	**Description**
				**(Cite year)**	
0	28	0.941	colorectal endoscopic submucosal dissection	2011	a minimally endoscopic treatment for large superficial colorectal tumors
1	28	0.957	early gastric cancer	2016	malignancies confined to the gastric mucosa or the submucosa
2	25	0.945	large colorectal tumor	2006	colorectal tumor with a size which is difficult to treat by EMR (usually over 20 mm)
3	25	0.986	superficial colorectal tumor	2015	neoplasm limited to mucosal or submucosal invasion <1,000 μm from the muscularis mucosae
4	23	0.916	superficial esophageal (tumor)	2012	neoplasm confined to the esophageal mucosa or the submucosa

* Size: the number of references that a cluster contains.

# Silhouette: the parameter indicates in efficiency of the clusters. The results were considered to be efficient and convincing, when the silhouette value is over 0.7.

** LLR: log-likelihood ratio.

The top ranked article by co-citation counts (Table 5) was Pimentel-Nunes P (2015) in Cluster #1, with citation counts of 148. The second one was Tanaka S (2015) in Cluster #3, with citation counts of 125,followed by Chung IK (2009) in Cluster #8, with citation counts of 97. Among the top 5 publications on ESD, three were regional clinical guidelines (Table 5).

**Table 5:** Top 5 co-citation references related to ESD, 2006-2020.

**Ranking**	**Frequency**	**Centrality**	**Source**	**Cited reference**	**Representative author (publication year)**	**Cluster**
1	148	0.24	Endoscopy	Endoscopic submucosal dissection: European Society of Gastrointestinal Endoscopy (ESGE) Guideline	Pimentel-Nunes P (2015)	1
2	125	0.17	Digestive Endoscopy	JGES guidelines for colorectal endoscopic submucosal dissection/endoscopic mucosal resection	Tanaka S (2015)	3
3	97	0.04	Gastrointestinal Endoscopy	Therapeutic outcomes in 1,000 cases of endoscopic submucosal dissection for early gastric neoplasms: Korean ESD Study Group multicenter study	Chung IK (2009)	8
4	95	0.24	Gastrointestinal Endoscopy	Advantage of endoscopic submucosal dissection compared with EMR for early gastric cancer	Oka S (2006)	6
5	94	0.05	Gastric Cancer	Japanese classification of gastric carcinoma: 3rd English edition	Japanese Gastric Cancer Association (2011)	8

### Journals

Table 6 lists the top 10 highly cited journals. The impact factors (IF) of the top 10 journals ranged from 3.665 to 23.059 (average of 8.818), with an IF >5.000 in 7 journals. The highest one was Gastrointestinal Endoscopy, with 1,900 citations (IF, 2020 = 9.472), followed by the Endoscopy (1,810 citations, IF, 2020 = 10.093) and Digestive Endoscopy (1,383 citations, IF, 2020 = 7.559). The top 3 journals are all official journals of the American Society for Gastrointestinal Endoscopy (ASGE), the European Society of Gastrointestinal Endoscopy (ESGE) and the Japan Gastroenterological Endoscopy Society (JGES), respectively.

**Table 6:** Top 10 highly cited journals in research of ESD, 2006-2020.

**Ranking**	**Journal**	**Frequency**	**Centrality**	**Impact factor (2020)**
1	Gastrointestinal endoscopy	1,900	0.43	9.427
2	Endoscopy	1,810	0.21	10.093
3	Digestive endoscopy	1,383	0.19	7.559
4	Surgical endoscopy	1,261	0.14	4.584
5	Gut	1,070	0.03	23.059
6	Gastric cancer	900	0.07	7.370
7	World journal of gastroenterology	865	0.03	3.665
8	American journal of gastroenterology	713	0.02	10.864
9	Journal of gastroenterology and hepatology	704	0.03	4.029
10	Journal of gastroenterology	659	0.04	7.527

### Keywords cluster analysis and burst detection

The analysis of keywords can be used to determine the hotspots in the literature. The top 10 keywords with the highest frequencies were endoscopic submucosal dissection, mucosal resection, resection, cancer, early gastric cancer, tumor, risk factor, outcome, EMR and efficacy (Supplementary Table 1). Keyword co-occurrence cluster analysis was visualized in Supplementary Figure 1.

**Figure 6:**
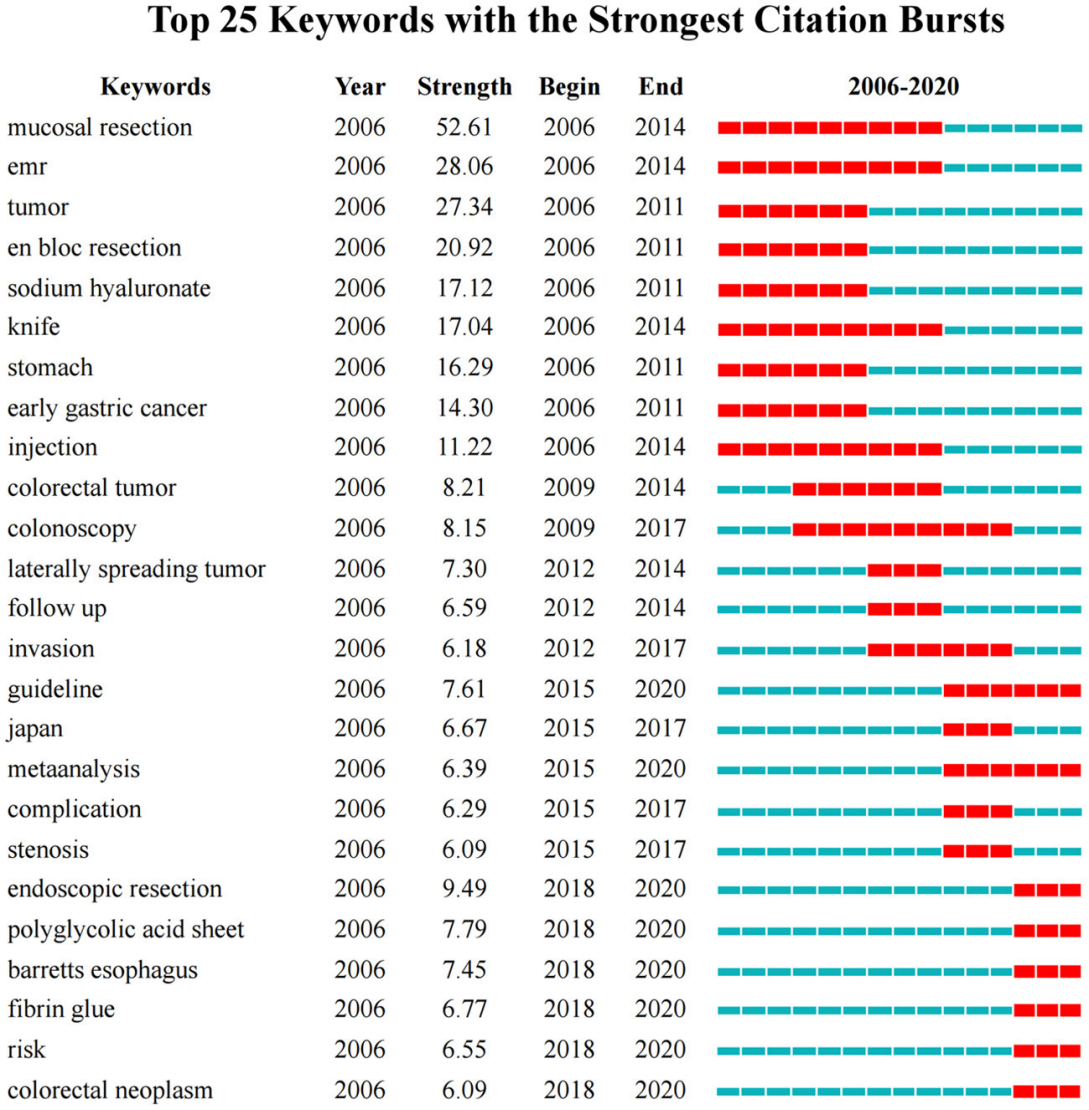
Keywords with the strongest citation bursts in published articles on ESD, 2006-2020. The timeline is depicted as a blue line, and the time interval that a subject was found to have a burst is shown as a red segment which indicated the beginning year, the ending year, and the duration of the burst.

The keywords are generalizations of the topics in the literature. Keywords burst detection can identify fast growing topics that last for multiple years as well as a single year in a specific research field (30). The top 25 keywords with the strongest citation bursts in publications on ESD were shown in Figure 6. Mucosal resection was the strongest burst keyword appeared from 2006 to 2014 with the burst strength of 52.61, followed by EMR (endoscopic mucosal resection) from 2006 to 2014 (28.06), tumor (27.34) from 2006 to 2011, and en bloc resection (20.92) from 2006 to 2011. Eight frontiers that have impacts on future research on ESD were guideline, meta-analysis, endoscopic resection, poly-glycolic acid sheet, Barrett's esophagus, fibrin glue, risk, and colorectal neoplasm.

## Discussion

In 1998, Hosokawa and Yoshida from Japan reported a new endoscopic mucosal resection procedure for early gastric cancer by using an insulation-tipped diathermic knife (IT knife), which could provide a one piece resection for a large lesion (1). Over the next few years, resection techniques that utilize direct dissection of the submucosa with a modified needle knife were developed and classified as endoscopic submucosal dissection (ESD) (2, 3). ESD has the advantage of permitting en bloc and histological complete resection regardless of lesion size. Evidence based on large cohort studies have confirmed its high en bloc and curative resection rates, as well as excellent long-term outcomes for early cancers and large superficial lesions in gut (11–14, 16, 31). Following the development of requisite devices and establishment of appropriate perioperative methods, the technical difficulties and adverse events rates of ESD have been greatly reduced. ESD has been recommended by updated guidelines from different countries/regions as a superior option for the treatment of gastrointestinal superficial neoplastic lesions, particularly for large ones (4–6, 15, 32). In the present study, we aimed to observe the development and detect the trends and frontiers in the research of ESD. Given the fact that few data were published (only 12 articles in English from 2001-2005) before 2006 when the technique was not well-developed, we choose the time span from 2006 to 2020 (15-years interval) for our analyses. It should be noted that the quantity of published literatures began to continually increase from 2014. As a technique originated in Eastern Asia, the role of ESD in treating superficial neoplastic lesions in GI tract was not established in early stage, until in 2014, the long-term effectiveness of the technique was confirmed by Pimentel-Nunes P (16), which may promote the further studies on ESD in Western countries from that time.

The top 10 prolific countries/regions consist of countries/regions from Asia, Europe, America, and Oceania, indicating ESD has been widely adopted in the world. The top 3 countries which accounted for 83.7% of the publications all came from Eastern Asia. Japan ranked top both in terms of number of publications and centrality, which indicated its most outstanding contribution to this field over the last 15 years. ESD was first reported by Japanese endoscopists (1–3). This advanced endoscopic resection technique had been well-developed and evaluated in Japan, including the procedure, requisite devices and clinical outcomes (11, 12, 14, 33). As the neighbors of Japan, by taking the advantage of distance to cooperate and communicate, South Korea and China ranked second and third respectively in the most prolific countries/regions list. There was a wide gap of publication volume and centrality between western and eastern countries. This unbalance may result from underestimating the need and benefit from ESD, relatively low incidence of suitable lesions for the procedure and lack of opportunities for proper training in the west (15, 16, 32).

Regarding the top 10 prolific institutes in research of ESD, Japan was the biggest contributor (7 institutes), followed by South Korea (3 institutes). It is obvious that all the active institutes were from Japan and its nearby countries, including South Korea and China, indicating the leading position of Eastern Asian countries in this field.

Cooperative relationship between authors/institutes was visualized in the present study, which can help to investigate and build potential partnerships. Our results showed that Japan was the biggest contributor to both the most productive authors and institutes in research of ESD. Professor Yutaka Saito, the Director of Endoscopy Division, National Cancer Center Hospital, Tokyo, was the most productive researcher in this field. Most of his influential publications focused on colorectal ESD, including the effectiveness, feasibility, safety and outcome (12, 34–37). He reported a series of data based on large numbers of colorectal ESDs (including the largest one involving 1,111 cases by far), providing strong evidence for its advantage in treating large superficial colorectal tumors (12, 34, 35, 38). The most influential author was Professor Naohisa Yahagi, the Director of Division of Research and Development for Minimally Invasive Treatment, Cancer Center, Keio University School of Medicine, Tokyo. He was one of the endoscopists who reported the first application of ESD for resection of esophageal neoplasms (39, 40). He also participated in the first study to report the clinical follow-up of colorectal ESD (41). These data provided valuable experience in the early stage of the development of this technique. The closer cooperation between Japanese authors (Figure 4) promoted high-quality multicenter cohort studies, providing reliable evidence for clinical guidelines (12, 42–48). 8 out of the top 10 prolific authors were members of the ESD Guidelines Committee of JGES. With 2 of the top 3 prolific authors, National Cancer Center Hospital, Tokyo became the most productive and influential research institution in this field. The features of connection lines (Figure 3) reveal domestic cooperation was the main mode of cooperation between institutes. International research cooperation on ESD needs to be strengthened in the future.

Reference co-citation relationship exists when two documents appear together in the bibliography of the third document. Co-citation analysis generates taxonomy of research, providing a knowledge base in a specialized field (49). Among the five main clusters in the co-citation network, 4 were main indications for ESD, including early gastric cancer, large colorectal tumor, superficial colorectal tumor and superficial esophageal tumors. As the superiority of the technique has been well-studied and confirmed, ESD is considered as the first-line therapy for these lesions meeting the indication criteria of the procedure (4–6, 32). It was also notable that 3 out of the 5 main clusters relate to colorectal ESD. Up to now, carrying out colorectal ESD is still technically more difficult than upper gastrointestinal ESD caused by the anatomical and histological characteristics of the colorectal wall. Folds and flexions in the colorectal tract make it more difficult to maneuvering the endoscope in the lumen, and the thinner colonic wall contributes to the higher risk of perforation rate. A relatively long learning curve in training also limits the wider acceptance of this technique worldwide. In addition, there is no evidence-based consensus on actual follow-up methods and time of surveillance after colorectal ESD (4). All these situations above may make researchers pay more attentions on colorectal ESD to facilitate the procedure and improve its safety and effectiveness.

The top co-cited literatures are often considered as the fundamental or basis for a certain research field. The top 2 and the fifth of top 5 co-citation references were guidelines in this field (32, 50). These publications provide recommendations in ESD treatment and a systematic review of evidence based on well-designed clinical studies, which play instructive roles in helping endoscopists detect the unsolved problems and raise future directions in the research of ESD. The remaining 2 publications were retrospective cohort studies on gastric ESD reported by Chung IK and Oka S (11, 13). Based on large samples of cases (1,000 and 1,020 early gastric cancers, respectively), these convincing results confirmed the safety and effectiveness, as well as the therapeutic advantages of ESD, which could be recognized as the cornerstones in the early developmental stage of this technique.

Highly cited journals reflect the important research sources in a certain field. In the present study, the top 3 highly cited journals in research of ESD were top journals in the field of endoscopy. Gastrointestinal Endoscopy, Endoscopy and Digestive Endoscopy, as the official journals of the Society for Gastrointestinal Endoscopy form different parts of the world (United States, Europe, and Japan, respectively), standing the leading roles in ESD research, which also indicate the well-established technique has been addressed and accepted worldwide. In addition, most journals in the top 10 highly cited list were highly influential ones in *Gastroenterology & Hepatology*, such as Gut, American Journal of Gastroenterology, Gastric Cancer and Journal of Gastroenterology, indicating this revolutionary technique has a great impact on endoscopic resection procedures and becomes one of the key topics with great interest in the field of endoscopic treatment.

According to the keyword co-occurrent analysis, some of the most important hotspots in this field were detected. Keywords with the most occurrent frequencies indicated the resection technique, indications, safety and efficacy have become the main research directions of ESD research in the past 15 years, which are also key issues for the development of clinical technique. This is consistent with the keyword co-occurrence clusters analysis, with “early gastric cancer”, “esophageal squamous cell neoplasm” and “esophageal cancer” as the target lesions, “proton pump inhibitor”, “pocket-creation method” and “conventional flushknife-bt” as the regimen/technique to improving perioperative safety, and “long-term outcome” as the evaluation of effectiveness.

The burst keywords detected by CiteSpace are potentially useful in predicting research frontiers (24). Based on the evolution of keywords bursts, the course of ESD development and its research present situation, as well as the future research trends could be concluded as follows.

As a therapeutic endoscopy firstly developed from EMR of EGC, earlier studies on ESD mainly focused on gastric superficial neoplasms, the advantage of en bloc resection when compared with EMR procedure. A series of studies based on large cases provided clear evidence for the positive therapeutic value of ESD, which became the cornerstones of the following research (11, 13, 14, 51). In subsequent years, with the accumulation of experiences of gastric ESD, the application of the procedure extended to colorectal lesions, which is more technically difficult. Studies on colorectal ESDs increased, particularly focusing on large lesions such as lateral spreading tumors (LST) which is most suitable for en bloc resection by ESD (12, 34, 42, 52). Meanwhile, in order to improve the curative resection rate and indication criteria, studies on exploring an accurate method of preoperative diagnosis of lesion invasion depth and the feasibility for resecting lesions with submucosal invasion were conducted (53–55). Data to evaluate the clinical outcomes based on short or long time follow-up of the previous ESD cases also increased (16, 48, 56). With a plenty number of studies on a specific topic, a series of meta-analysis were induced, which contributed, together with well-designed clinical studies, to the establishment practical guidelines on ESD.

Although the advantages of ESD have been well-established, it is still a technique with high risks. Post-ESD bleeding occurs as an adverse event which is not well-prevented with methods of standard care. No consensus has been reached on risk factors of postoperative bleeding (57, 58). Although application of polyglycolic acid (PGA) sheets and fibrin glue was reported to be promising in preventing post-ESD bleeding, the efficacy of such shielding method is still controversial (59, 60), which need to be addressed in further study.

It is notable that Barrett's esophagus has become a hotspot in this field. As the lesion with a high risk of esophageal adenocarcinoma, there is discrepancy in the pathological evaluation of Barrett's esophagus-related neoplasms between Eastern and Western countries, due to the different definition and the discordance of the incidence, especially for long-segment Barrett's esophagus (LSBE). By now, diagnosing lateral extent of the cancer before ESD is still difficult and the rate of R0 resection is not satisfactory (6). Therefore, it will be also one of the research trends in the future.

Lastly, up to date, compared with the quantity of clinical research, data on ESD from animal study is limited. ESD on living piglets have been reported in the application of endoscopists training and evaluation the new techniques (61, 62). Taking the advantages of animal experiments, porcine model will be a useful tool in establishment of training standards and exploring technical innovations for ESD in the future.

Our study is the first application of bibliometric analysis in research on ESD. However, the limitation of the study is the lack of inclusion of other public and commercially available bibliometric databases, such as Scopus, Medline, and PubMed which might be difficult to perform co-citation analysis due to the lack of information on cited references. In addition, studies of other languages were also not included in the analysis. These limitations above might result in selection bias. Bibliometric research based on multiple databases and different languages will be needed to furtherly evaluate the research status of ESD.

In conclusion, the bibliometric results in our study provide a clear visual analysis of the quantity, quality, citations and keywords of studies on ESD over the past 15 years. With a rapid increasing number of publications, ESD are receiving an extensive attention by endoscopists worldwide. Our findings may help clinicians and scientists discover the status, possible collaborators and emerging trends of ESD research.

## Data availability statement

The raw data supporting the conclusions of this article will be made available by the authors, without undue reservation.

## Author contributions

MW and DW designed the study. MW, DW, and MJ performed the analysis and interpreted the data. DW and MJ edited the manuscript. MW, SZ, and XX reviewed the article and provided comments or suggestions. MW: had primary responsibility for final content. All authors contributed to the article and approved the submitted version.

## Funding

This study was supported by the General Program of National Natural Science Foundation of China (NSFC, Grant No. 81970554). The funders had no role in the conduct of the study, the analysis or interpretation of data, and the preparation, review, or approval of the manuscript.

## Conflict of interest

The authors declare that the research was conducted in the absence of any commercial or financial relationships that could be construed as a potential conflict of interest.

## Publisher's note

All claims expressed in this article are solely those of the authors and do not necessarily represent those of their affiliated organizations, or those of the publisher, the editors and the reviewers. Any product that may be evaluated in this article, or claim that may be made by its manufacturer, is not guaranteed or endorsed by the publisher.
