# Supplementary material for: Studies on endoscopic submucosal dissection in the past 15 years: A bibliometric analysis
Source: Front Public Health. 2022 Sep 27;10:1014436. doi: 10.3389/fpubh.2022.1014436 (PMC9552180; doi:10.3389/fpubh.2022.1014436)
Supplement: Supplementary file 1 [file Table_1.DOCX]

**Supplementary Table 1. Top 10 keywords with the highest occurrence frequency** **of ESD research, 2006-2020**

| Rank | Keywords | Count | Centrality |
| --- | --- | --- | --- |
| 1 | endoscopic submucosal dissection | 846 | 0.02 |
| 2 | mucosal resection | 639 | 0.06 |
| 3 | resection | 496 | 0.10 |
| 4 | cancer | 456 | 0.05 |
| 5 | early gastric cancer | 414 | 0.03 |
| 6 | tumor | 329 | 0.10 |
| 7 | risk factor | 269 | 0.09 |
| 8 | outcm (outcome) | 245 | 0.02 |
| 9 | emr | 237 | 0.05 |
| 10 | efficacy | 216 | 0.11 |
