# Supplementary figures and images for: Studies on endoscopic submucosal dissection in the past 15 years: A bibliometric analysis
Source: Front Public Health. 2022 Sep 27;10:1014436. doi: 10.3389/fpubh.2022.1014436 (PMC9552180; doi:10.3389/fpubh.2022.1014436)

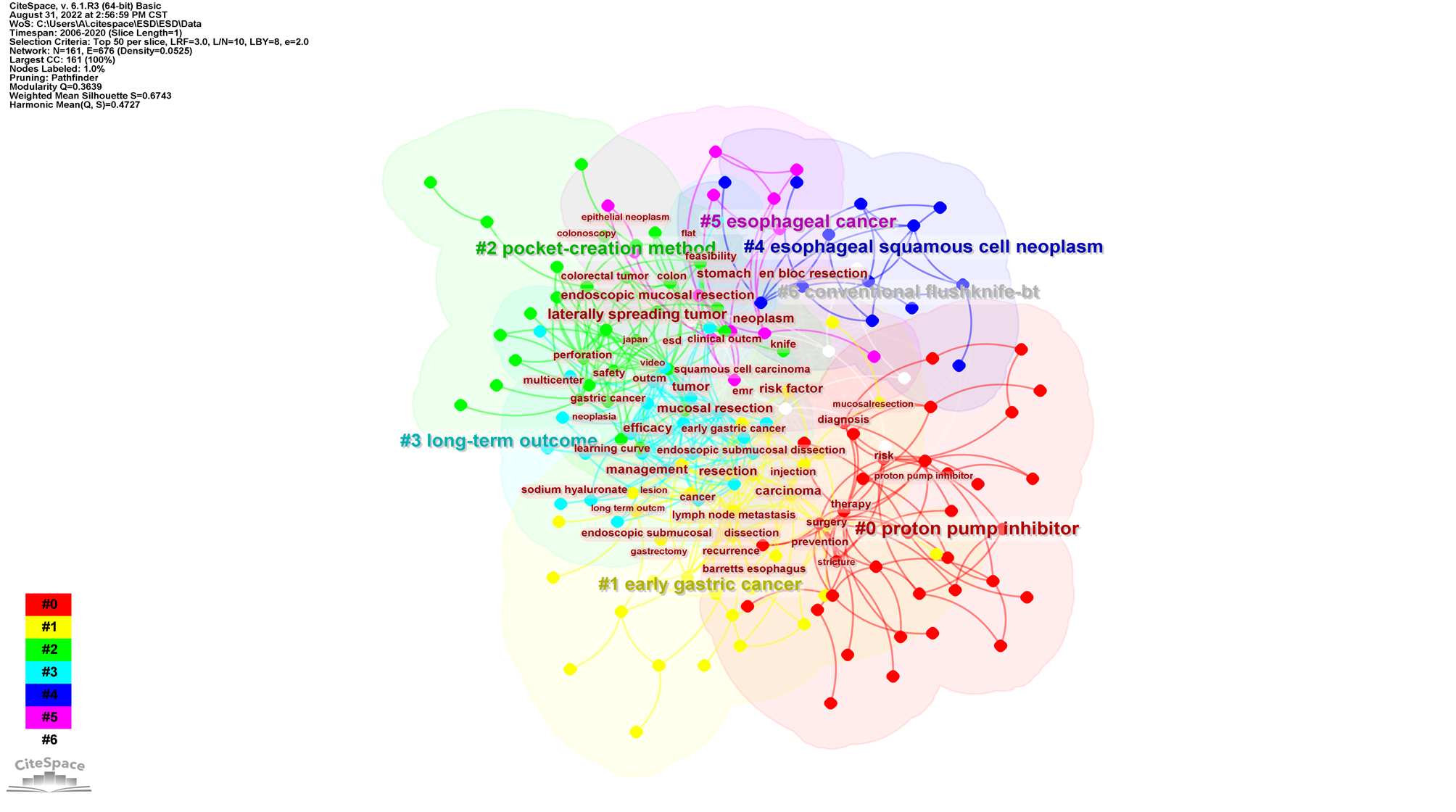

Supplement: Supplementary file 2 [file Image_1.TIF]
